# Phosphorus uptake and toxicity are delimited by mycorrhizal symbiosis in P-sensitive *Eucalyptus marginata* but not in P-tolerant *Acacia celastrifolia*

**DOI:** 10.1093/aobpla/plac037

**Published:** 2022-08-21

**Authors:** Mark Tibbett, Matthew I Daws, Megan H Ryan

**Affiliations:** Department of Sustainable Land Management and Soil Research Centre, School of Agricultural Policy and Development, University of Reading, Reading, Berkshire RG6 6AR, UK; School of Biological Sciences, The University of Western Australia, 35 Stirling Highway, Crawley, WA 6009, Australia; Department of Sustainable Land Management and Soil Research Centre, School of Agricultural Policy and Development, University of Reading, Reading, Berkshire RG6 6AR, UK; School of Agriculture and Environment, The University of Western Australia, 35 Stirling Highway, Crawley, WA 6009, Australia

**Keywords:** *Acacia*, fertilizer, mycorrhiza, P accumulation, P-use efficiency, P toxicity, rehabilitation, restoration

## Abstract

Many plant species from regions with ancient, highly weathered nutrient-depleted soils have specialized adaptations for acquiring phosphorus (P) and are sensitive to excess P supply. Mycorrhizal associations may regulate P uptake at high external P concentrations, potentially reducing P toxicity. We predicted that excess P application will negatively impact species from the nutrient-depleted Jarrah forest of Western Australia and that mycorrhizal inoculation will reduce P toxicity by regulating P uptake. For seedlings of the N_2_-fixing legume *Acacia celastrifolia* and the tree species *Eucalyptus marginata*, we measured growth at P concentrations of 0–90 mg kg^−1^ soil and in relation to inoculation with the arbuscular mycorrhizal fungus (AMF) *Rhizophagus irregularis*. Non-inoculated *A. celastrifolia* maintained leaf P concentrations at <2 mg g^−1^ dry mass (DM) across the range of external P concentrations. However, for non-inoculated *E. marginata*, as external P concentrations increased, leaf P also increased, reaching >9 mg g^−1^ DM at 30 mg P kg^−1^ soil. *Acacia celastrifolia* DM increased with increasing external P concentrations, while *E. marginata* DM was maximal at 15 mg P kg^−1^ soil, declining at higher external P concentrations. Neither DM nor leaf P of *A. celastrifolia* was affected by inoculation with AMF. For *E. marginata*, even at 90 mg P kg^−1^ soil, inoculation with AMF resulted in leaf P remaining <1 mg g^−1^ DM, and DM being maintained. These data strengthen the evidence base that AMF may not only facilitate P uptake at low external P concentrations, but are also important for moderating P uptake at elevated external P concentrations and maintaining plant P concentrations within a relatively narrow concentration range.

## Teaser

Arbuscular mycorrhizal fungi (AMF) are typically thought to facilitate phosphorous (P) uptake when external P concentrations are low. However, for two species adapted to low-nutrient environments, we investigated whether AMF can also control and reduce P uptake at high external P concentrations. For one of the species, *Eucalyptus marginata*, we found evidence that AMF not only facilitate P uptake at low external P concentrations but are also important for moderating P uptake at elevated external P concentrations and maintaining plant P concentrations within a relatively narrow concentration range.

## Introduction

Ancient and highly weathered soils, such as those found in South West Western Australia, have naturally low phosphorus (P) concentrations. Many plants adapted to growth under these low P conditions have evolved a range of strategies for P acquisition, including cluster roots and exudation of carboxylates and phosphatases (Lambers *et al*. 2006, 2008). The majority of plant taxa from one of many mycorrhizal symbiotic types (Kariman *et al*. 2018), many of which serve to exploit soil nutrients through mycelia scavenging of resources not available to roots alone (Tibbett 2000; Tibbett and Sanders 2002; Smith *et al*. 2015).

Species adapted to naturally low soil P concentrations may display symptoms of P toxicity when supplied with P concentrations above those that they experience naturally in soil (Handreck 1991; Lambers *et al*. 2002; Shane *et al*. 2004b; Standish *et al*. 2007; Pang *et al*. 2010; de Campos *et al*. 2013; Williams *et al*. 2019), due potentially to the loss of low-affinity transporter systems (Huang *et al*. 2011). P-sensitive species occur in a range of families, including the Fabaceae, Haemodoraceae, Myrtaceae, Proteaceae and Rutaceae. Symptoms of P sensitivity are highly species-specific and occur at shoot P concentrations less than 1 mg g^−1^ dry mass (DM) to more than 40 mg P g^−1^ DM (Shane *et al*. 2004a and references therein). Symptoms of P toxicity include a reduction in growth with increasing external P concentration (e.g. Standish *et al*. 2007; Williams *et al*. 2019), and visible symptoms including early leaf senescence and necrotic and chlorotic regions on leaves (e.g. Handreck 1991; Lambers *et al*. 2002; Shane *et al*. 2004a, b; Kariman *et al*. 2014a; Ye *et al*. 2021). 

Mycorrhizal symbioses are well known for increasing P uptake in nutrient-deficient soils and increasing the P status of host plants (Bougher *et al*. 1990; Koide and Mosse 2004; Smith *et al*. 2011; Kariman *et al*. 2018; Standish *et al*. 2022). However, they can also enable the growth of plants in soils containing toxic concentrations of heavy metals, or certain essential trace elements such as cadmium and zinc, by controlling the uptake of metal ions (Jentschke and Godbold 2000; Hildebrandt *et al*. 2007; de Oliveira *et al*. 2020; Yazici *et al*. 2021). Similarly, AMF can modify P uptake in the host plant by downregulating the expression of genes encoding high-affinity phosphate transporter proteins (Burleigh and Bechmann 2002). While mycorrhizal associations can increase plant shoot P concentrations by increasing uptake at low P availability and enabling exploitation of a greater soil volume (Tibbett 2000), there is increasing evidence for AMF and ectomycorrhizal fungi (ECM) (Kariman *et al.* 2014a) that they can also moderate shoot P at high P availability. For example, Nazeri *et al*. (2014) demonstrated, for a range of legume species, that inoculation with AMF could maintain shoot P concentrations within relatively narrow boundaries following the application of a single pulse of P. This effect was modulated by both mycorrhizal-related reductions in rhizosphere carboxylates and P transport from roots to shoots. Conversely, high P availability can itself inhibit both formation of arbuscules and intraradical colonization (e.g. Müller and Harrison 2019).

Jarrah (*Eucalyptus marginata*) is a dominant overstorey tree in the Jarrah forest of South West Western Australia and, based on pot experiments, is known to be sensitive to elevated external P (Kariman *et al*. 2014a, 2016). For example, in the absence of inoculation with AMF, Kariman *et al*. (2014a) reported the onset of leaf chlorosis and necrosis in the week following application of a pulse of P to Jarrah seedlings. When seedlings were inoculated with AMF, visible symptoms associated with P toxicity and shoot P concentrations were reduced. While studies have largely focused on visible phytotoxicity symptoms resulting from P application (e.g. Handreck 1991; Lambers *et al*. 2002; Shane *et al*. 2004a; Kariman *et al*. 2014a), a recent study by Williams *et al*. (2019) demonstrated that P toxicity can also be expressed as a significant reduction in growth rates at shoot P concentrations that do not necessarily result in visible symptoms. Consequently, there is a need to better understand both longer-term effects of applying P and potential interactions of AMF on shoot P concentrations and plant growth.

For Jarrah, these observations in relation to P may also have significant practical implications: large areas of Jarrah forest are cleared and restored each year following bauxite mining. Fertilizer application (especially P), to maximize early plant growth, is generally viewed as a key step in the rehabilitation process (Ward *et al*. 1990; Grant *et al*. 2007; Tibbett 2010). However, there may be the potential for negative impacts on plant growth from applying excess P, particularly given newly restored forest has low species diversity and abundance of AMF (Gardner and Malajckuk 1988; Glen *et al*. 2008). Our own unpublished observations have found very poor levels of colonization by AMF (near absent) in seedlings of acacias and eucalypts in recently restored sites (M. Tibbett and M. H. Ryan, unpublished).

In the light of potential P toxicities in tree seedlings, and the prospect for symbiotic mitigation of such effects, we investigated two linked hypotheses anticipating contrasting responses for two species with distinctive ecological strategies: *Acacia celastrifolia* and *E. marginata. Acacia celastrifolia* is a large understorey ruderal legume that exhibits a strong growth response as a seedling in the field and Jarrah (*E. marginata*) is the dominant overstorey tree which constitutes around 80 % of stems in the native forest (Daws *et al*. 2015; Tibbett *et al*. 2020). Like many Australian *Acacia* spp., *A. celastrifolia* can form associations with AMF and N_2_-fixing rhizobia and *E. marginata* can form associations with both AMF and ECM (Reddell and Milnes 1992; Kariman *et al.* 2012). We undertook two experiments, the first one to assess the effect of P-application rate on growth of both species and the second to assess the effect of AMF inoculation on the response to P application. Based on these experiments we tested firstly the hypothesis that *A. celastrifolia* would exhibit a strong growth response to P addition over a wide range of exogenous supply, whereas Jarrah would not, and would potentially show signs of growth depression and toxicity at high P-application rates. Our second hypothesis was that AMF inoculation would alter the response in terms of P supply, growth and uptake, leading to a suppression in acacia growth and offer a remedial effect on Jarrah growth response and P uptake at high amendment rates.

## Materials and Methods

### Plant and soil material

Seeds of *E. marginata* (Jarrah) and *A. celastrifolia* were collected in the northern Jarrah forest of Western Australia, ca. 130 km SSE of the state capital Perth (32°48ʹS, 116°28ʹE). Seeds were collected from a minimum of five individual plants per species. *Eucalyptus marginata* is the dominant tree species and *A. celastrifolia* is an abundant and widespread understorey shrub in the Jarrah forest. Prior to experimentation, seeds were surface-sterilized in a 1:3 dilution of sodium hypochlorite solution for 10 min followed by repeated rinsing in distilled water. The water-impermeable seed coat of *A. celastrifolia* was chipped at the end furthest from the axis using a scalpel and seeds of both species were soaked for 2 h in 1:10 ‘Seed Starter’ smoke water (Kings Park Botanic Gardens and Parks Authority, Perth, WA). Seeds were sown on the surface of 10 % agar water and placed at 15 °C in the dark.

For both experiments, once seeds had commenced germination, seedlings were transplanted into sealed 9.6-L pots at a depth of 10 mm. The pots contained ca. 4 L of disinfested topsoil that had been steamed twice for 3 h at 80 °C, dried at 40 °C and then sieved to 4 mm. The topsoil used in the experiment was also obtained from the northern Jarrah forest of Western Australia (32°48ʹS, 116°28ʹE). Jarrah forest soils are gravelly with low concentrations of available N, P and K (Tibbett *et al*. 2020) and high rates of P fixation on amorphous iron and aluminium oxides. Soil available (Colwell) P concentrations are low, typically ~2–3 mg kg^−1^ soil (Tibbett *et al.* 2020; Daws *et al.* 2021). Ten germinated seeds were placed into each pot at a depth of 10 mm. For the second, mycorrhizal inoculation experiment, inoculum of *Rhizophagus irregularis* (formerly *Glomus intraradices*) was placed beneath the seeds at the time of planting. The inoculum was created using single species pot cultures from the University of Western Australia culture collection, originally sourced from INVAM (https://invam.wvu.edu/home). The AM inoculum consisted of *R. irregularis* hyphae and colonized leek (*Allium porrum*) roots, grown in sterilized river sand. To balance any potential edaphic changes that might occur from adding this culture material to the experimental pots containing Jarrah forest soil, autoclaved culture material was placed beneath the seeds in the non-mycorrhizal treatments. Pots were watered weekly to 50 % field capacity and seedlings were thinned to the two healthiest plants after 21 days, and then down to one plant after 31 days.

To ensure that only P was limiting in the experiment, and that no nutrient imbalances were induced by the addition of P, 10 mL of modified Long Ashton’s nutrient solution (minus P) was added to each pot 15 days after seedlings were planted (Cavagnaro *et al*., 2001). Macronutrients: K_2_SO_4_ (20 mM), MgSO_4_·7H_2_O (15 mM), CaCl_2_·2H_2_O (30 mM), FeEDTA (1 mM), (NH_4_)_2_SO_4_ (40 mM), NaNO_3_ (80 mM). Micronutrients: H_3_BO_3_ (28.6 mg L^−1^), MnCl_2_·4H_2_O (18.1 mg L^−1^), ZnSO_4_·7H_2_O (2.2 mg L^−1^), CuSO_4_·5H_2_O (0.8 mg L^−1^), NaMoO_4_·2H_2_O (0.25 mg L^−1^).

### Experimental design

Twenty-three days after planting, phosphate was added to the P treatments in the form of potassium dihydrogen phosphate (KH_2_PO_4_). To ensure a constant ionic background and balanced potassium levels, potassium chloride (KCl) was added in inverse proportions to KH_2_PO_4_ amendments. In Experiment 1, eight rates of P application were used (equivalent to 0, 0.9, 4.5, 13.5. 22.5, 31.5, 40.5 and 81 mg elemental P kg^−1^ soil) to assess the susceptibility of the two plant species to different external P concentrations. In Experiment 2 there were four P-application rates (equivalent to 0, 4.5, 30 and 90 P kg^−1^ soil) in a two-way factorial combination of P-application rate × mycorrhizal treatment to test the effect of AMF on P uptake at different external P concentrations. Both experiments were established in randomized blocks in a glasshouse, with each block containing all treatments. The temperature-controlled glasshouse was maintained at temperatures between 18 °C and 28 °C. Pots were regularly re-randomized throughout the growing period. All treatments were replicated three times for Experiment 1 and four times for Experiment 2. The levels of P-application rate were chosen to span the entire range of current and past fertilizer prescriptions applied in the rehabilitation of Jarrah forest ecosystems (Standish *et al.* 2015).

### Mycorrhizal activity (Experiment 2)

Thirteen days after transplanting and at the end of the experiment (188 days), seedlings were screened for evidence of colonization by AMF. Roots were cleared using KOH then stained using lactic-glycerol blue and examined by light microscopy for evidence of colonization (Brundrett *et al*. 1996). At the end of the experiment, AM spores were also extracted from 150 g of soil sampled away from the middle of the pots following wet sieving and sucrose centrifugation (Walker *et al*. 1982). Spores were extracted and counted (in addition to direct measures of colonization) as sporulation provides an indication of the overall level of mycorrhizal abundance and reproduction over the duration of the experiment.

### Plant measurements

For Experiment 1, plants were harvested 213 days after transplanting and roots and shoots dried separately at 70 °C for dry weight determination. For Experiment 2, plants were harvested for DM determination 53 or 188 days after transplanting. In both experiments, plants were carefully removed from the growing medium, roots washed with water and the plants separated into roots and shoots.

### Foliar P concentrations

Dried leaf material from the last sampling time points in both experiments was ground and then subsampled for digestion. Leaf material was digested using an HNO_3_/HClO_4_ mixture with the diluted digest approximately 10 % v/v with respect to HClO_4_ (70 % w/w). Phosphorus content was determined using the molybdovanadophosphate method (yellow) and a spectrometer reading at a wavelength of 460 nm (modified from Simmons 1975, 1978).

### Statistical analysis

For Experiment 1, two-way ANOVA implemented in Minitab 14 was used to assess, whether there were effects of (i) species and (ii) increasing external P concentration, on either plant DM or leaf P concentration. Data were log_10_ transformed. For Experiment 2, two-way ANOVA was used to assess, for each of the two species, the effect of (i) inoculation with AMF and (ii) external P concentration on either plant DM or leaf P concentration. Finally, for Experiment 2, for the plants of each of the study species inoculated with AMF, two-way ANOVA was used to assess the interaction of (i) study species and (ii) external P concentration on spore count in the soil at the end of the experiment. Spore counts were log_10_(*n* + 1) transformed to ensure normality.

## Results

### Experiment 1: Effect of external P concentration on DM and leaf P of non-inoculated plants

**Figure 1.**
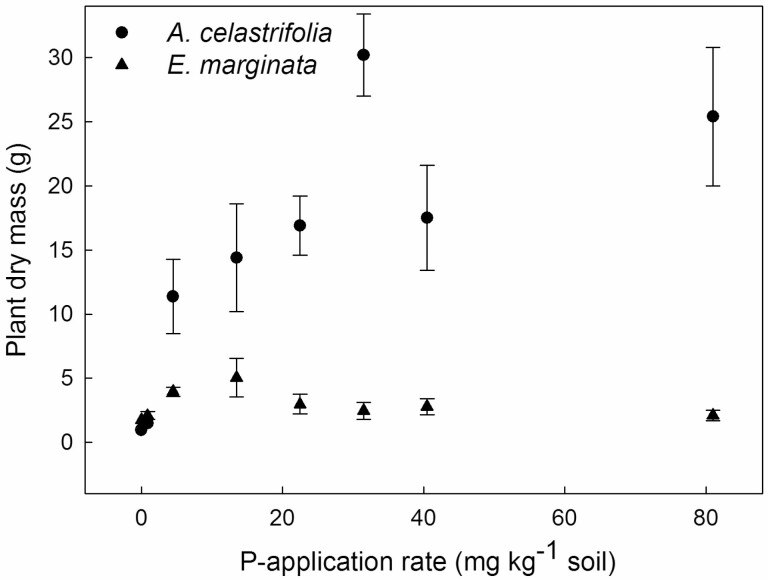
The effect of P-application rate on plant DM of non-AMF-inoculated *Acacia celastrifolia* and *Eucalyptus marginata* assessed 213 days after transplanting. Bars ± 1 SE.

For non-inoculated plants, there was a significant effect of P-application rate on plant DM after 213 days (two-way ANOVA, *F*_1, 32_ = 18.50, *P* < 0.001), but the two species (*A. celastrifolia* and *E. marginata*) responded differently to applied P (two-way ANOVA, *F*_1, 32_ = 107.20, *P* < 0.001; Fig. 1). Across the range of concentrations from 0 to 15 mg P kg^−1^ soil, *A. celastrifolia* DM increased rapidly with increasing P. At higher P-application rates, the rate of increase in DM declined. Nonetheless, total DM at the highest application rate (81 mg kg^−1^ soil) was 10× higher than at 0 mg P kg^−1^ soil (Fig. 1). For *E. marginata*, there was also an initial increase in DM as P-application rate increased from 0 to 15 mg kg^−1^ soil (Fig. 1). However, at P-application rates greater than 15 mg kg^−1^, DM declined: plant mass at a P-application rate of 15 mg kg^−1^ was more than twice that at 81 mg kg^−1^ (Fig. 1).

**Figure 2.**
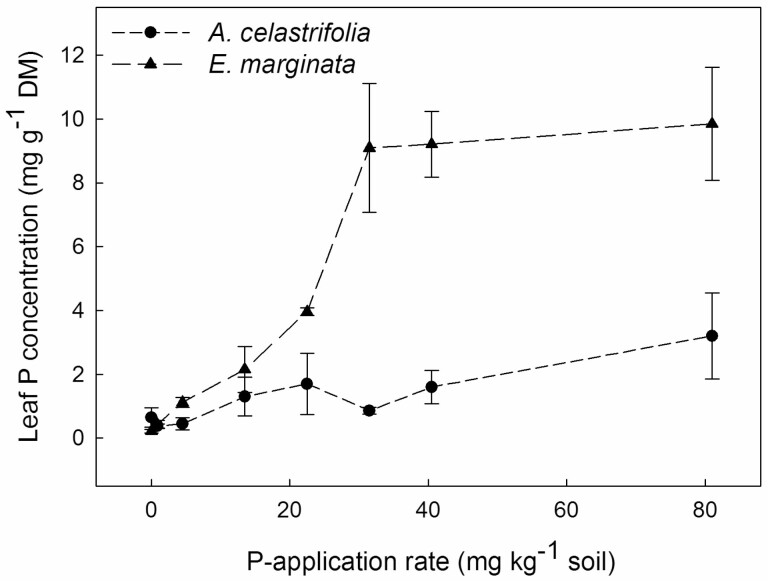
The effect of P-application rate on leaf P concentration for non-AMF-inoculated *Acacia celastrifolia* and *Eucalyptus marginata* assessed 213 days after transplanting. Bars ± 1 SE.

Leaf P concentrations of *A. celastrifolia* were ca. 0.65 mg g^−1^ DM for plants at the nil P-application rate, then increasing to a maximum of ca. 3.2 mg g^−1^ DM at the P-application rate of 81 mg kg^−1^ soil (Fig. 2). For *E. marginata* leaf P was ca. 0.22 mg g^−1^ DM at the nil P-application rate (Fig. 2) and increased rapidly with P application reaching ca. 9 mg g^−1^ DM at 30 mg P kg^−1^. As the P-application rate increased to 80 mg kg^−1^, leaf P concentrations remained at ca. 9 mg g^−1^ DM (Fig. 2). These responses were reflected in significant main effects of P-application rate (two-way ANOVA, *F*_7, 32_ = 19.58, *P* < 0.001) and species (two-way ANOVA, *F*_1, 32_ = 26.28, *P* < 0.001) as well as a significant P application × species interaction (two-way ANOVA, *F*_7, 32_ = 4.85, *P* = 0.001). For *E. marginata* the leaf P concentration corresponding to maximum plant DM accumulation, and above which DM accumulation declined, was ca. 4 mg g^−1^ DM (Figs 1 and 2).

### Experiment 2: Effect of AM colonization on DM and leaf P

Evidence of early colonization (6–29 %) by AM was found in 4 of the 12 samples taken (one *A. celastrifolia* and three *E. marginata*). Roots taken at the end of the experiment did not clear properly and were not able to be assessed reliably and consequently were discarded. There was no evidence of colonization by ECM for either plant species, nor was there evidence of nodulation in *A. celastrifolia*. Spores were found only in the mycorrhizal treatments, and levels of sporulation varied consistently with P-application rates, supporting the use of sporulation as an acceptable proxy for measuring mycorrhizal activity. Spore counts from mycorrhizal pots were low (under 100 per 150 g soil) for both *A. celastrifolia* and *E. marginata* at the nil P-application rate, whereas no spores were found in non-inoculated treatments. There was a significant effect of plant species on sporulation in the inoculated treatment (two-way ANOVA, *F*_1, 24_ = 11.64, *P* < 0.01) with sporulation varying greatly between the species at the nil P-application rate, peaking at 4.5 mg kg^−1^ P for *E. marginata* yet remaining fairly low at all P-application rates (Table 1). In contrast, sporulation continued increasing with P application for *A. celastrifolia*, peaking at 90 mg kg^−1^ P with a mean of 276 spores per 150 g of soil. This difference in response between the two species was reflected in the P-application rate × species interaction being highly significant (two-way ANOVA, *F*_3, 24_ = 9.34, *P* < 0.001), but the main effect of P application being non-significant (two-way ANOVA, *F*_3, 24_ = 1.91, *P* = 0.154).

**Table 1.** The effect of plant species, P-application rate and inoculating pots at the outset of the experiment with the AMF *Rhizophagus irregularis* on spore counts at the end of the experiment. Spore counts were taken at the end of Experiment 2 on Day 188. Error bars are ± 1 SE of the mean.

Plant species	P-application rate (mg kg^−1^ soil)	Spore count (pots inoculated with AMF) (# spores 150 g^−1^ soil)	Spore count (pots not inoculated with AMF) (# spores 150 g^−1^ soil)
*Acacia celastrifolia*	0	45.3 ± 4.4	0
	4.5	117.3 ± 13.0	0
	30	191.8 ± 27.0	0
	90	276.0 ± 52.5	0
*Eucalyptus marginata*	0	80.5 ± 3.5	0
	4.5	103.3 ± 9.0	0
	30	58.5 ± 5.8	0
	90	37.0 ± 4.3	0

**Figure 3.**
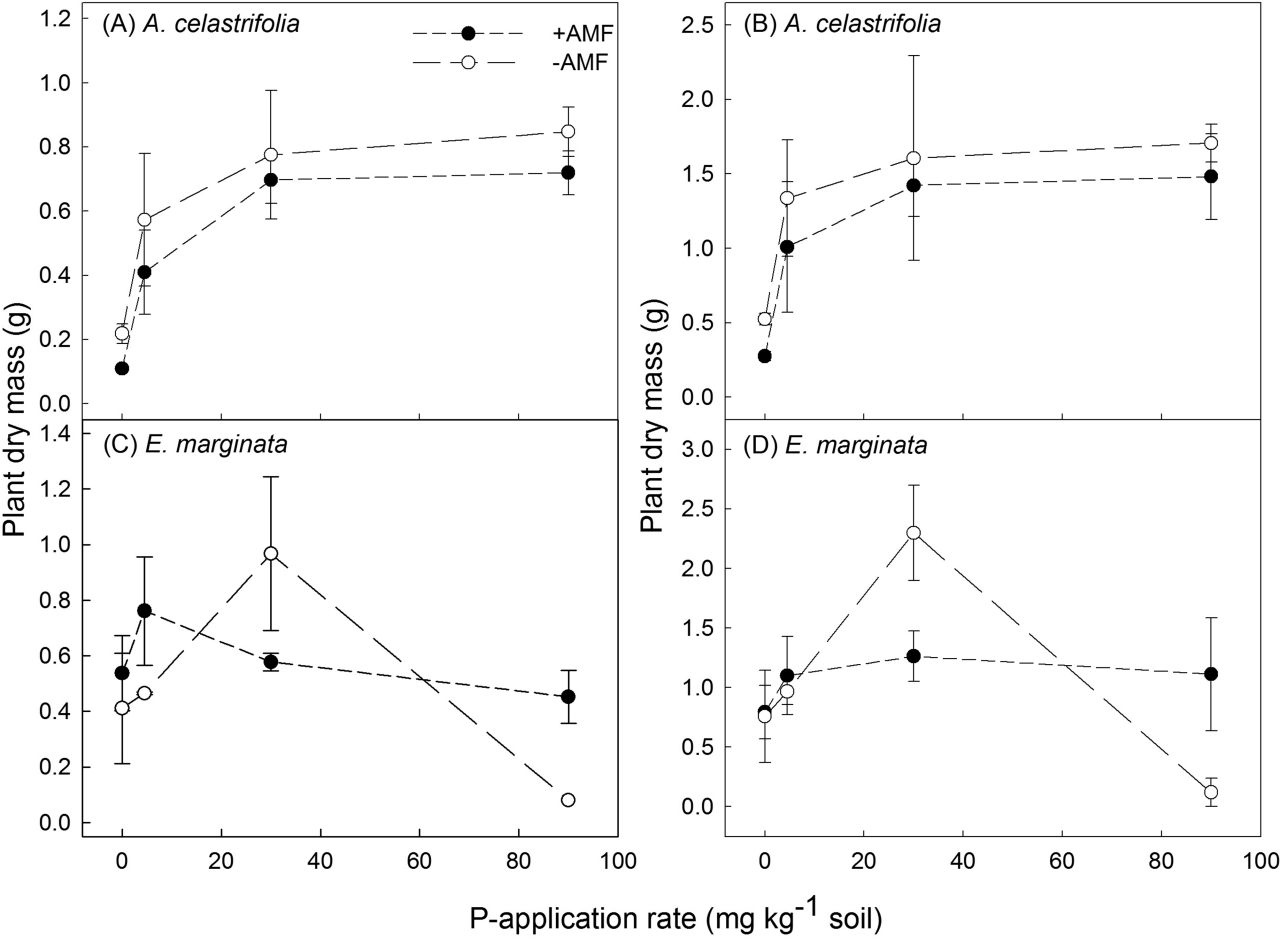
The effect of P-application rate and inoculation with the AMF *Rhizophagus irregularis* on plant DM assessed either 53 (A and C) or 188 days after transplanting (B and D) for *Acacia celastrifolia* and *Eucalyptus marginata.* Note that due to elevated mortality at high P, the 53-day data point for *E. marginata* at 90 mg P kg^−1^ consists of data from one plant only. Bars ± 1 SE.

For both plants inoculated and non-inoculated with AMF, for the two time periods that were measured (53 and 188 days), there was a significant effect of P-application rate on DM for *A. celastrifolia* (two-way ANOVA, *F*_3, 24_ = 11.0, *P* < 0.001 and *F*_3, 24_ = 4.94, *P* < 0.01 for 53 and 188 days, respectively) and *E. marginata* (two-way ANOVA, *F*_3, 24_ = 3.92, *P* < 0.05 and *F*_3, 24_ = 4.32, *P* < 0.05 for 53 and 188 days, respectively) (Fig. 3A and B). *Acacia celastrifolia* DM increased with P-application rate and the rate of increase declined above a P-application rate of 30 mg kg^−1^ soil. For *A. celastrifolia*, there was no effect of AM inoculation on DM accumulation for either measurement interval (Fig. 3A and B; two-way ANOVA, *F*_1, 24_ = 1.96, *P* = 0.175 and *F*_1, 24_ = 1.01, *P* = 0.324 for 53 and 188 days, respectively).

At both measurement intervals, DM of non-inoculated *E. marginata* plants increased reaching a maximum at the P-application rate of 30 mg kg^−1^ soil and declined thereafter (Fig. 3C and D). At a P-application rate of 90 mg kg^−1^ soil, there were visible symptoms of P toxicity including leaf necrosis. Further, for the replicate sampled at 53 days after planting, only one of the four replicate plants was still alive. For plants inoculated with AMF, there was no effect of P-application rate on DM accumulation. Further, neither a decline in DM nor visible symptoms of P toxicity were observed at the highest P-application rate of 90 mg P kg^−1^ soil (Fig. 3C and D). At the second measuring interval these responses were reflected in a significant P-application rate × AMF inoculation interaction (two-way ANOVA, *F*_3, 24_ = 3.62, *P* < 0.05). Profound differences between inoculated and non-inoculated plants can be seen in Supporting Information—Fig. S1.

**Figure 4.**
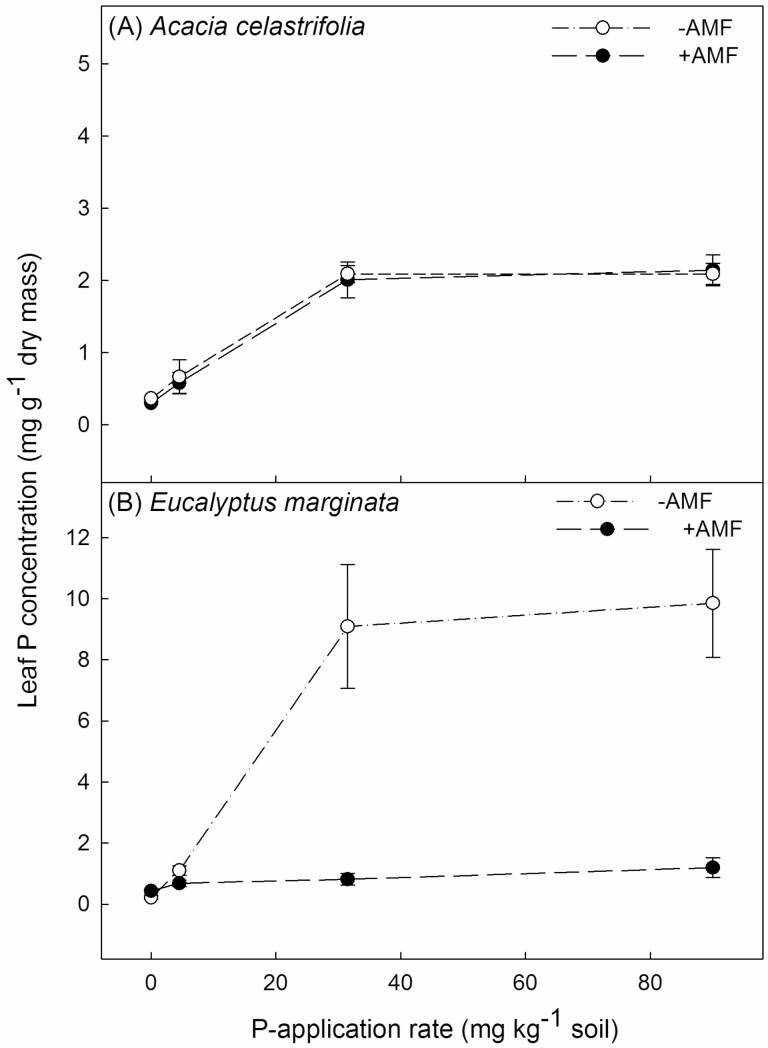
The effect of P-application rate and inoculation with the AMF *Rhizophagus irregularis* on leaf P concentration for *Acacia celastrifolia* and *Eucalyptus marginata* assessed 188 days after transplanting. Bars ± 1 SE.

Leaf P concentration of *A. celastrifolia* increased from ca. 0.6 to 2 mg g^−1^ DM as the P-application rate increased from 0 to 90 mg kg^−1^ soil with this response being significant (two-way ANOVA, *F*_3, 24_ = 63.77, *P* < 0.001). This response was independent of inoculation with AMF (Fig. 4A; two-way ANOVA, *F*_1, 24_ = 0.14, *P* = 0.71). For non-inoculated *E. marginata* plants, the leaf P concentration increased from ca. 0.2 to 9 mg g^−1^ DM as the P-application rate increased from 0 to 90 mg kg^−1^ soil (Fig. 4B). However, for plants of *E. marginata* inoculated with AMF, leaf P concentration did not respond to an increasing P-application rate and remained at ca. 0.5 mg g^−1^ DM across the entire range of P-application rates which was reflected in a significant P-application rate × AM inoculation interaction term (two-way ANOVA, *F*_3, 22_ = 7.66, *P* < 0.001; Fig. 4B).

## Discussion

For non-inoculated plants of *A. celastrifolia*, an increasing P-application rate increased DM up to high-level application rates (80 mg P kg^−1^ soil). However, for non-inoculated plants of *E. marginata*, an increasing P supply increased growth only at relatively low external P concentrations: thereafter DM declined with increasing P supply. Similar contrasting patterns in response to increasing P supply have been observed previously for a range of Australian species from severely nutrient-impoverished environments (Grundon 1972; Groves and Keraitis 1976; Handreck 1997; Pang *et al*. 2010; de Campos *et al*. 2013, Williams *et al*. 2019). For *E. marginata*, but not *A. celastrifolia*, inoculation with AMF reduced growth at moderate P supply but facilitated growth at high P supply by regulating leaf P concentrations.

For non-inoculated *E. marginata*, maximum DM was observed at P-application rates of 15–30 mg kg^−1^ soil, before declining at higher P-application rates. Maximum DM of a range of Australian natives has been reported to occur across a similar range of P supply (e.g. Bougher *et al*. 1990; Ryan *et al*. 2009; Pang *et al*. 2010; Williams *et al*. 2019). For example, for the 11 species studied by Pang *et al*. (2010), maximum growth occurred at P-application rates in the range 12–24 mg P kg^−1^. Further, for 8 of the 11 species, growth declined at P-application rates greater than those required for maximum plant DM. Shane *et al*. (2004a) reported that, for a range of species, P toxicity occurred at leaf P concentrations of 0.9–47 mg g^−1^ DM. The leaf P concentrations at which we observed negative effects on growth of *E. marginata* (>4 mg g^−1^ DM) are at the lower end of these reported values. However, Williams *et al*. (2019) reported for *Eucalyptus torquata* that a reduction in growth occurred at leaf P concentrations > 2 mg g^−1^ DM. Possible explanations for the differences between our current values and those reported by Shane *et al*. (2004a) are that either there are a wide range of species-specific thresholds for the onset of toxicity or that the values reported by Shane and co-workers are thresholds for the onset of *visible* symptoms of P toxicity (e.g. necrosis). It is worth noting that our results and those of Williams *et al*. (2019) indicate the onset of a negative effect on plant growth and not necessarily the onset of visible symptoms.

Leaf P concentrations of non-inoculated *A. celastrifolia* initially increased with increasing P supply. However, even at higher P supply, leaf P concentrations did not exceed ca. 2 mg g^−1^ DM. Similarly, Williams *et al*. (2019) reported that *Acacia acuminata* exhibited an initially increasing shoot P concentration in response to increasing P supply, but as P-application rates increased further, shoot P concentration was maintained at ca. 2 mg g^−1^ DM. Conversely, *Acacia hemiteles* exhibited increasing shoot P with increasing P supply: shoot P reached concentrations of ca. 8 mg g^−1^ DM, and growth reduced as shoot P concentration continued to increase (Williams *et al*. 2019). de Campos *et al*. (2013) also reported on the ability of two *Acacia* species (*Acacia truncata* and *Acacia xanthina*) to regulate internal P concentrations in relation to external P concentrations. *Acacia truncata* was unresponsive in terms of DM accumulation as the external P concentration increased, whilst *A. xanthina* exhibited declining DM as P increased. These results suggest that the growth response to elevated P, even within co-occurring members of a genus, can be unpredictable as also reported for the genus *Banksia* (de Campos *et al*. 2013).

We found low levels of early colonization by AMF in both species in 4 of the 12 samples examined. Similarly, low levels of colonization have been reported in previous studies with Jarrah forest species. For example, Kariman *et al*. (2012) reported that although evidence of colonization by AMF of *E. marginata* in a pot experiment was found in as few as 2.3 % of samples, beneficial effects of AMF were still observed. Interestingly, even at low rates of intraradical colonization, AMF can still modulate the expression of phosphate transporters (Bulgarelli *et al*. 2020; Chu *et al*. 2020). Further, it should be noted that root colonization is not necessarily required for positive physiological responses in plant–fungus interactions (Neumann 1959; Kariman *et al*. 2014b). Similar results have also been reported for ECM colonization on seedlings of *Eucalyptus diversicolor* where in the absence of applied P, despite colonization rates on roots ranging from only 1 to 6 %, there was still a significant growth benefit for seedlings resulting from inoculation (Bougher *et al*. 1990).

For *A. celastrifolia*, plant DM was consistently, but not significantly, lower in the AM inoculation treatment across the entire range of P-application rates. While AMF can increase P uptake and result in increased growth (Smith *et al*. 2015), the transfer of carbon from the host plant to the AMF can also result in negative effects on plant growth. Indeed, this is further implied by the increased level of AMF sporulation observed at high P concentrations when in association with *A. celastrifolia*. In comparison, inoculation with AMF had three contrasting effects on DM of *E. marginata*.

First, at a low P-application rate (4.5 mg P kg^−1^), at 53 days after planting, there was a positive classical growth effect of inoculation on DM. While inoculation with AMF did not increase leaf P concentrations it had increased total P uptake. Similarly, Kariman *et al*. (2014a) reported for 14-week-old *E. marginata* seedlings grown under P-deficient conditions that inoculation with the AMF *R. irregularis* did not increase leaf P concentration. In contrast, at low external P concentrations Jones *et al*. (1998) reported that inoculation with AMF both increased shoot P concentrations and growth of *Eucalyptus coccifera*.

Second, at a moderate P-application rate (30 mg kg^−1^), inoculation with AMF significantly depressed growth suggesting that at this supply, the association was parasitic rather than mutualistic. Indeed, for a range of species at elevated P concentrations, associations with AMF have been shown to move from mutualistic to parasitic (Johnson *et al*. 1997; Hoeksema *et al*. 2010; Johnson 2010).

Third, at high P-application rates, inoculation with AMF significantly increased DM, compared to non-inoculated plants, whilst maintaining leaf P concentrations within a similar range to that observed at lower external P concentrations. This is not a classical plant growth effect, rather a suppression of toxicity due to the symbiosis. We posit this mechanism is either related to a (down)regulation of root epidermal transporters which has been observed in AM plants for P, and at high concentrations for cadmium and putatively arsenic (De Oliveira *et al*. 2020; Kariman *et al*. 2014a; Nazeri *et al*. 2014; Kariman *et al*. 2016) or to a loss of function of the direct P-uptake pathway into roots colonized by AMF (Smith *et al.* 2003). Interestingly, for Jarrah, this effect of P-toxicity attenuation is occurring at high P availability when it is likely that there is increasing negative regulation of the AM symbiosis (e.g. Müller and Harrision 2019) which is supported by the declining spore counts we observed at high P. In nature, where plants are commonly mycorrhizal (Kariman *et al*. 2018), this may be a common mechanism whereby plants are protected from toxicities (at least to some extent) by mycorrhizal symbiosis.

Except for our control, nil P treatment, leaf P concentrations for both of our study species when non-inoculated were higher than concentrations previously reported in plants growing in relatively undisturbed and unfertilized Jarrah forest. For example, values of 0.4–0.45 mg P g^−1^ DM have been reported for *E. marginata* (Hingston *et al*. 1981, M. I. Daws, unpublished data) and 0.3 mg P g^−1^ DM for *A. celastrifolia* (M. I. Daws, unpublished data). For *E. marginata* inoculated with AMF, leaf P was maintained at concentrations similar to those observed in unfertilized forests across the full range of P-application rates. P is widely applied to newly established *E. marginata* stands following post-mining rehabilitation (Standish *et al.* 2015) and a recent study demonstrated significant ongoing impacts of applying P at the outset of rehabilitation on the re-establishing Jarrah forest plant community. Specifically, applied P shifts the balance between different plant functional groups favouring fast-growing legumes compared to long-lived understorey resprouter species (Daws *et al.* 2021) suggesting that P should be applied with caution. These results may have broader application to the use of fertilizer for rehabilitation of other biodiverse plant communities growing on P-deficient soils elsewhere (e.g. Tibbett *et al*. 2019). For rehabilitated Jarrah forest, investigating the role of AMF on plant growth and P uptake in the field would be of value, particularly since colonization of roots in newly established sites may be limited by the availability of propagules, e.g. spores, hyphae, colonized roots (Jasper *et al*. 2007). Arbuscular mycorrhizal fungus colonization could be manipulated, for example, by inoculating plants prior to planting in the field to act as nurse plants that accelerate the development of common mycorrhizal networks, facilitating fitness in plant species with high mycorrhizal dependency (Kariman *et al*. 2012; Montesinos-Navarro *et al*. 2019; Sortibrán *et al*. 2019; Standish *et al*. 2022).

Arbuscular mycorrhizal fungi are generally viewed as being important for increasing P uptake and facilitating growth at low external P concentrations. However, our data support a growing understanding that by regulating plant P concentration within a sufficient concentration range, AMF play an important role at high external P concentrations in enabling plant growth at concentrations that would otherwise result in reduced growth and P toxicity.

## Supporting Information

The following additional information is available in the online version of this article—

Figure S1. Photograph of Jarrah (*Eucalyptus marginata*) seedlings from Experiment 2 (see Materials and Methods section) grown at a P-application rate of 90 mg kg^−1^ soil. The pot on the left was not inoculated, whereas the pot on the right was inoculated with the arbuscular mycorrhizal fungus *Rhizophagus irregularis* at the commencement of the experiment. The photograph was taken at the end of the experiment (Day 188 after transplanting).

plac037_suppl_Supplementary_Figure_Legend

plac037_suppl_Supplementary_Figure_S1

## Data Availability

Data are available at https://doi.org/10.17864/1947.000399.

## Notes

**Form & Function. Chief Editor:** Kate McCulloh
