# Supplementary material for: Phosphorus uptake and toxicity are delimited by mycorrhizal symbiosis in P-sensitive Eucalyptus marginata but not in P-tolerant Acacia celastrifolia
Source: AoB Plants. 2022 Aug 21;14(5):plac037. doi: 10.1093/aobpla/plac037 (PMC9521482; doi:10.1093/aobpla/plac037)
Supplement: plac037_suppl_Supplementary_Figure_Legend [file plac037_suppl_supplementary_figure_legend.docx]

**Supplementary material**

**Figure S1.** Photograph of Jarrah (*Eucalyptus marginata*) seedlings from **experiment 2** (see methods section) grown at a P-application rate of 90 mg kg^-1^ soil. The pot on the left was not inoculated whereas the pot on the right was inoculated with the arbuscular mycorrhizal fungus *Rhizophagus irregularis* at the commencement of the experiment. The photograph was taken at the end of the experiment (day 188 after transplanting).
