# Supplementary figures and images for: Phosphorus uptake and toxicity are delimited by mycorrhizal symbiosis in P-sensitive Eucalyptus marginata but not in P-tolerant Acacia celastrifolia
Source: AoB Plants. 2022 Aug 21;14(5):plac037. doi: 10.1093/aobpla/plac037 (PMC9521482; doi:10.1093/aobpla/plac037)

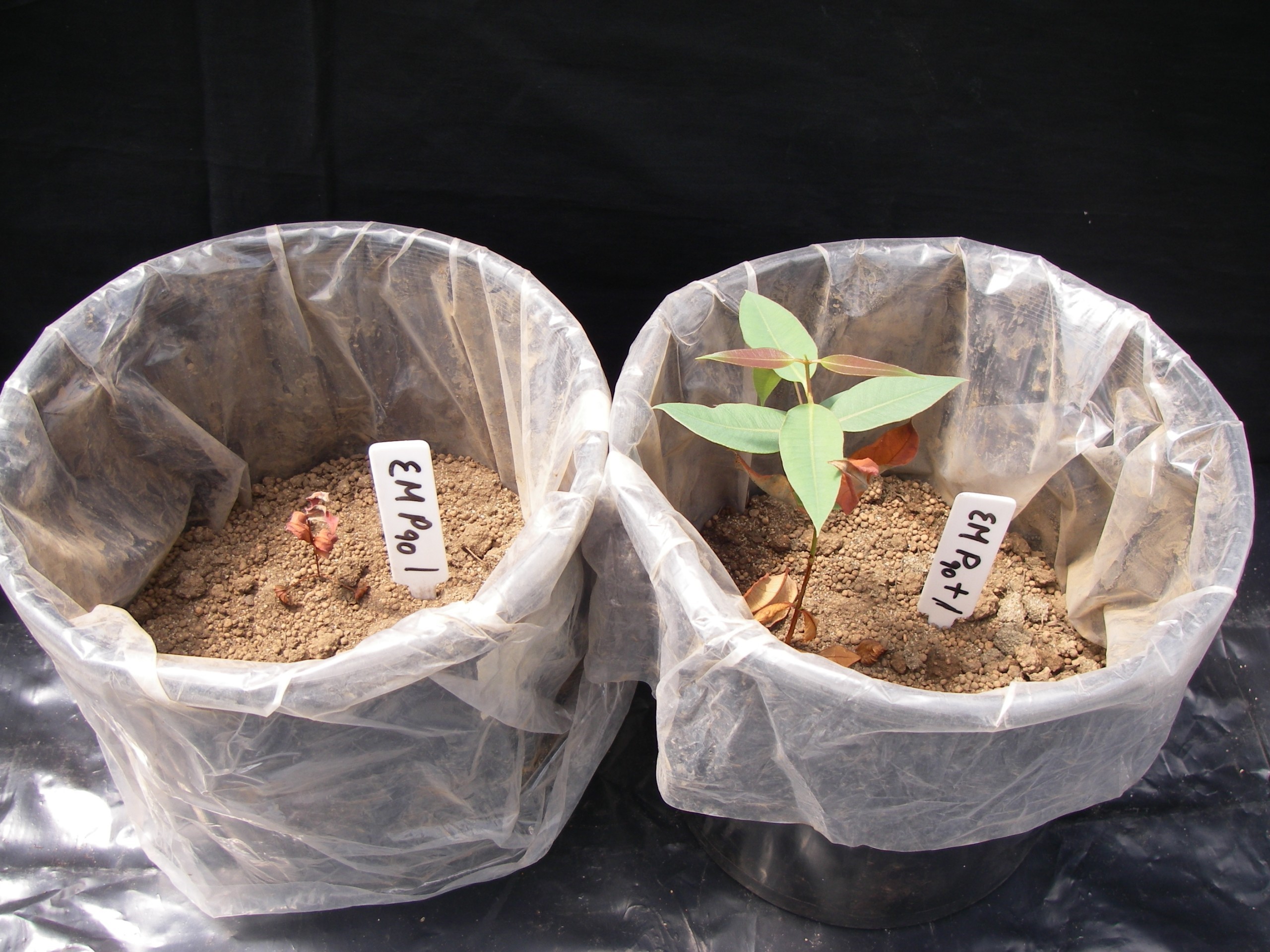

Supplement: plac037_suppl_Supplementary_Figure_S1 [file plac037_suppl_supplementary_figure_s1.jpeg]
